# Topical administration of Metamizole and its implications on vascular reactivity in Wistar rats- Experimental research

**DOI:** 10.22336/rjo.2017.6

**Published:** 2017

**Authors:** Ioana-Cristina Coman, Horia Paunescu, Alina Cristina Stamate, Alina Popa Cherecheanu, Isabel Ghita, Cosmina Barac, Danut Vasile, Ruxandra Tudosescu, Ion Fulga

**Affiliations:** * Pharmacology and Pharmacotherapy Department, ”Carol Davila” University of Medicine and Pharmacy, Faculty of Medicine, Bucharest, Romania; ** Ophthalmology Department, Emergency University Hospital, Bucharest, Romania; *** Surgery Department, Emergency University Hospital, Bucharest, Romania

**Keywords:** Metamizole, vascular reactivity, iris, long posterior ciliary artery

## Abstract

**Aim:** The aim of this paper was to describe the possible implications of topical (ocular) administration of Metamizole on vascular reactivity of the iris in Wistar rats. No other study regarding its topical use was found.

**Methods:** Male adult Wistar rats were anaesthetized with Ketamine 100 mg /kg body weight - injected intraperitoneally - while maintaining spontaneous respiration and the blink reflex. After selecting the area of interest (long posterior ciliary artery – LPCA), manual adjustments of the image magnitude, clarity, and brightness were made, and the experiment began. The image recording lasted 10 minutes.

**Results:** Metamizole induced a slight vasoconstriction that started with the initial moment for all the doses used. After the topical administration of Metamizole, we did not observe an increase of the vascular diameter of LPCA in a dose dependent manner. The saline solution used as a negative control did not modify the vessel diameter.

**Conclusions:** Metamizole (dipyrone) is a non-opioid drug, which is commonly used in human and veterinary medicine. It is the most popular first-line analgesic in various populations. In some cases, this agent is still incorrectly classified as a non-steroidal anti-inflammatory drug. The high analgesic efficacy of metamizole, as well as its spasmolytic effect, makes it a very important pharmaceutical agent that could be used in the therapy of various eye disorders in humans and in animals.

**Abbreviations:** COX = Cyclooxygenase; LPCA = Long Posterior Ciliary Artery; PRP = panretinal photocoagulation; PDR = proliferative diabetic retinopathy; Sec = second(s); VSPR = very severe non proliferative diabetic retinopathy

## Introduction

Metamizole is a pro-drug, which spontaneously breaks down to structurally related pyrazolone compounds after oral administration. Besides its analgesic effect, Metamizole is an antipyretic and spasmolytic agent. The mechanism responsible for the analgesic effect is complex and most probably based on the inhibition of a central cyclooxygenase-3 and the activation of the opioid system and the cannabinoid system. The mechanism responsible for the spasmolytic effect of metamizole is associated with the inhibited release of intracellular Ca2+ as a result of the reduced synthesis of inositol phosphate [**1**]. All the studies in the ophthalmological field focused on its effectiveness in reducing pain after laser procedures (panretinal photocoagulation- PRP) [**2**,**3**]. For example, it has been described that the use of 1000 mg of metamizole 40 min before PRP significantly reduces the pain associated with proliferative diabetic retinopathy (PDR) and very severe non proliferative diabetic retinopathy (VSPR) [**2**].

The aim of this paper was to describe the effects of Metamizole on iris vascular bed after the topical (ocular) administration in Wistar rats. It is important to mention that Metamizole (dipyrone) is commonly used in human and veterinary medicine; no other study regarding its local/ topical use was found.

## Materials & Methods

Adult male Wistar rats, weighing 250 g to 350 g, were used for the experiments and were brought in the laboratory facilities with a minimum of three days before the experiments began, being kept on a standard diet, with water and food supply ad libitum. All the experiments were performed during daytime (9:00 AM to 6 PM), and conducted in a noise-attenuated environment. All the animal procedures were carried out with the approval of the Local Ethics Committee of “Carol Davila” University of Medicine and Pharmacy, Bucharest, Romania, in accordance with the European Community Council Directive 86/609/EEC on the protection of animals used for scientific purposes. 

**Fig. 1:**
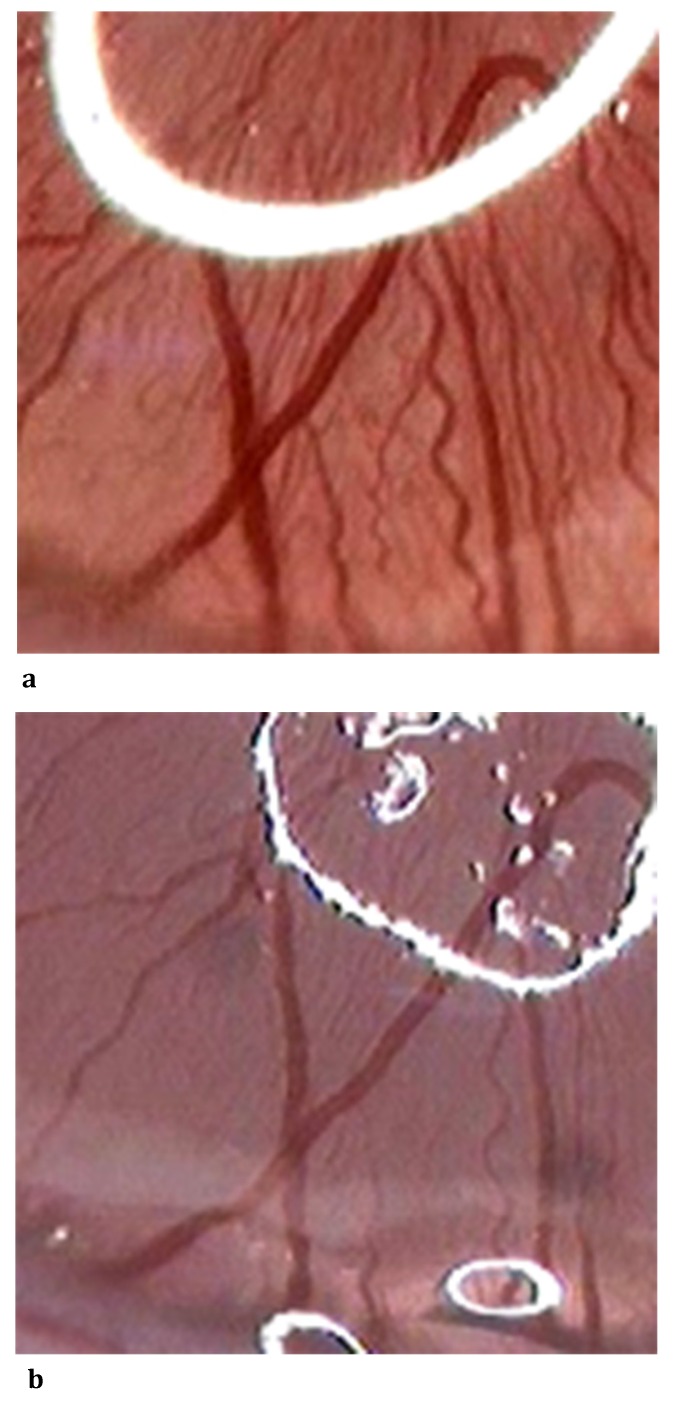
The aspect of LPCA diameter before and after topic administration of Metamizole 5%; a-Vascular diameter at T0i (0 sec); b- Vascular diameter at T4 (600 sec); we could observe a visible vasoconstriction between the initial and the final moment

The substances used were: Ketamine 5% (Calypsol 50mg/ml produced by Gedeon Richter PLC HU), Pancuronium Bromide Hospira (GB), Metamizole sodium monohydrate (Algocalmin solution 1g/ 2ml produced by Zentiva) and sodium chloride 0.5%. All rats were anaesthetized with Ketamine 5% -100 mg/ kg body weight - injected intraperitoneally - while maintaining spontaneous respiration and the blink reflex; after five minutes, Pancuronium Bromidum 0.02%, 0.1 mL/ 100 g body weight –injected intraperitoneally - was used to induce myorelaxation. Data recording was started after 10 minutes. After selecting the area of interest (long posterior ciliary artery – LPCA), manual adjustments of the image magnitude (maximum 400 X), clarity, and brightness were made, and the experiment began. The image recording lasted 10 minutes; two instillations at 30 and 330 seconds were used. The test solutions were applied topically without touching the ocular surface. The temperature of substances instilled was 37°C. The first drug was saline (sodium chloride 0.5%), the second one was the active substance (Metamizole 2.5%, 5%, 10% and equimolar doses of Metamizole 3.33%, 6.66%, 13.33%). Each subject served as his own control. The experiment design was parallel. The number of rats per group was 6, testing only the right eye (see **Fig. 1**).

The image acquisition system was composed of a CCD camera (Toshiba IK–642E) and an AD converter interface (Pinnacle microVideo DC10+) connected to an ASUS PC compatible system. The camera was fitted with a magnifying objective (Nikon) aided by an adapter (Navitar 1X Adapter 1–6015), allowing for resolutions within the optical microscopy range. Cold light was provided by a circular (ring-type fiber optics) source (Dolan–Jenner Industries Inc. model FiberLite series 180). The camera was mounted on a holder (produced by IOR, Romania) allowing it to focus on the eye of the animal. The maximum optical resolution attained by the system was 12400 dpi (a pixel representing around 2×2 micrometers). After immobilizing the animal, the optical system was adjusted manually until the image on the screen was adequately rich in blood vessels and its clarity was optimal. For maximum accuracy, the lighting conditions and the adjustment of the optical system were kept constant during the recording. To avoid the possible capture of image errors, the image adjustment, and acquisition was made for a single iris vascular area for each animal.

**Fig. 2:**
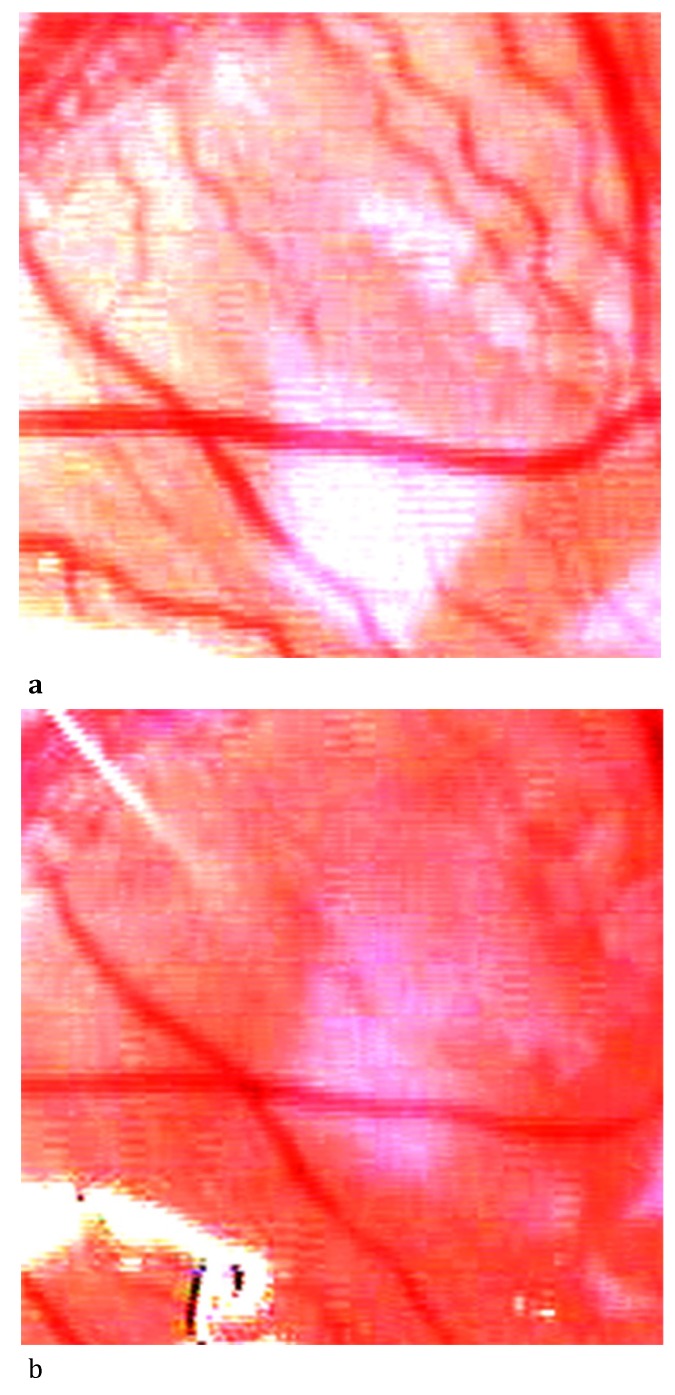
The aspect of LPCA diameter before and after topic administration of Metamizole 6.66%; a- Vascular diameter at T0i (0 sec); b- Vascular diameter at T4 (600 sec)

The image analysis was carried out by using VirtualDub and Adobe PhotoShop CS6 (see **Fig. 1** and **2**) and by measuring the variations of the vessels diameters before and after topic administration, at fixed time intervals: 0(T0i), 30(T1i), 120(T2i), 210(T3i), 300(T0), 330(T1), 420(T2), 510(T3), 600(T4) seconds (so nine different measurements were made for each eye). The first value of vessels diameter, at 0 seconds (D0i) was considered as control value for each eye registration. Five diameters (mm) were measured at equidistant intervals of 10 pixels and the average value and standard deviation value were calculated for each of the 5 vascular diameters. Microsoft Excel was used for statistical processing of data. The comparison was made solely at the same target area according to the initial conditions at 0s and 300s (e.g. T1i vs T1, T2i vs T2, T3i vs T3, T4i vs T4.) Thereby, the relative variations of the vascular diameter were calculated. 

Actual values (Da) were analyzed in relation with the initial value (D0i) by the following formulas: 

Vrel = (Da-Di0)/ Di0*100

For all the 6 rats, the Vrel. values and the mean standard error were analyzed by using the T-test.

## Results

**Fig. 3:**
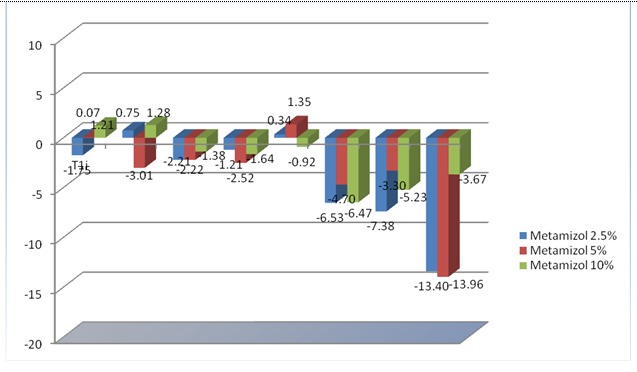
Relative variation (based on T0i value) of the vascular diameter of LPCA when using Metamizole 2.5%, 5% and 10%. Each value represents the percent of variation of vascular diameter at 30s (T1i), 120s (T2i), 210s (T3i), 300s (t4i), 330s (T1), 420s (T2), 510 s (T3), 600s (T4) and standard error

**Fig. 4:**
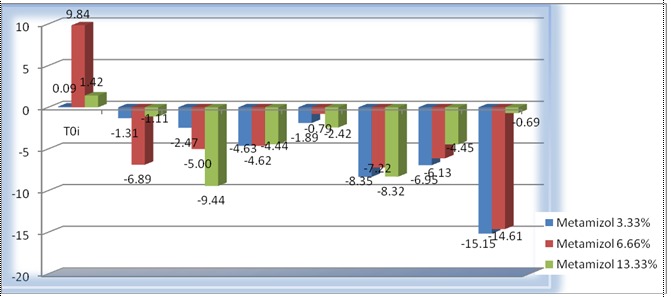
Relative variation (based on T0i value) of the vascular diameter of LPCA when using Metamizole 2.5%, 5% and 10%. Each value represents the percent of variation of vascular diameter at 30s (T1i), 120s (T2i), 210s (T3i), 300s (t4i), 330s (T1), 420s (T2), 510 s (T3), 600s (T4) and standard error

The results were presented in **Fig. 1**-**4**.

The saline solution used as a negative control did not modify the vessel diameter. We could not recognize a pattern of vasodilation or vasoconstriction during the first 5 minutes of our recordings.

As it was seen in **Fig. 3** and **4**, Metamizole induced a slight vasoconstriction that started with the initial moment for all doses used; a significant vasoconstriction was obtained only for the medium doses used (5% and 6.66%) and only for the final moment (T4). A statistical significance for Metamizole 5% was obtained at the final moment (T4) (p=0.04) and for Metamizole 6.66% at T4 (p=0.02); also, for Metamizole 10% a tendency towards vasoconstriction at T2 (420 sec) was observed, the values being at the limit of the statistical significance (p=0.053).

## Discussion

This particular experimental model was chosen to observe the local effects of the topical administration of Metamizole on vascular reactivity. The reason for this choice was the diversity of effects Metamizole provides and the lack of similar studies from literature. Also, its systemic reactions are partially known, but there is no research made on its local effects associated with topical administration. Metamizole is a relatively safe pharmaceutical preparation although it is not completely free from undesirable effects. Among these side effects, the most serious one that raises most controversy is the myelotoxic effect. It seems that in the past, the risk of metamizole-induced agranulocytosis was exaggerated. Despite the evidence showing no risk of teratogenic and embryotoxic effects, the drug must not be administered in pregnant women, although it can be given to pregnant and lactating animals. The mechanism of action of Metamizole, especially the one responsible for the analgesic effect, is complex [**1**,**4**]. Although for years it has been claimed to be part of non-steroidal anti-inflammatory drugs (NSAIDs), apart from its analgesic effect, the medication has antipyretic and spasmolytic effects as well. The drug produces only a very weak anti-inflammatory effect, which is most probably the consequence of its weak inhibition on cyclooxygenase1 (COX-1) and 2 (COX-2) [**5**].

The mechanism responsible for the analgesic effect of Metamizole most probably rests on the inhibition of a central cyclooxygenase-3 and activation of the opioidergic system and cannabinoid system; the mechanism responsible for the spasmolytic effect is associated with the inhibited release of intracellular Ca2+ as a result of the reduced synthesis of inositol phosphate [**1**,**6**].

The topical administration of drugs is the most preferred route for the management of eye disorders because it provides higher ocular drug concentrations, avoiding powerful systemic side effects associated with oral administration [**7**]. Considering that Dipyrone is detectable in the serum for only about 15 min following the intravenous administration, and it is not detectable after oral intake [**5**], a considerable part of its side-effects can be avoided when using topical pathway.

Our findings suggested a tendency towards the vasoconstriction on iris territory, probably via prostaglandin-induced vasoconstrictor tonus (at the level of LPCA), COX1-mediated, the latter being the main functional enzyme in the platelets which couples preferentially, with thromboxane synthase [**8**]. Some studies suggested that the analgesic effect of dipyrone may be partly mediated by a dual mechanism of action: the inhibition of COX enzyme activity and the stimulation of CB receptors [**5**,**9**]. Thus, further studies should focus on the neuropathic pain from Dry Eye Syndrome and the effects of topical Metamizole. Local side-effects of topical ophthalmic NSAIDs, which are often used in Dry Eye Syndrome, include transient burning, stinging, conjunctival hyperemia and corneal anesthesia [**10**].

According to relatively recent reports, a more severe complication involves the association of topical ophthalmic NSAIDs with indolent corneal ulceration and full-thickness corneal melting [**10**]. There might be many risk factors for NSAIDs-associated adverse reactions in diverse populations. Autoimmune diseases, such as and Sjögren’s syndrome, bacterial infections, rheumatoid arthritis, chronic dry eye syndrome and rosacea are common disorders associated with corneal ulcer-formation [**11**]. On the other hand, the side effects of NSAIDs are preferred over those of opioids, whose sedative and anesthetic effects could interfere with ocular homeostasis. That being considered, the use of topical Metamizole could exclude or diminish the numerous side effects of these two types of drugs and improve the outcome in those particular cases. For example, a comparative study that focused on the preemptive analgesia associated with the oral administration of metamizole versus ibuprofen in patients going through retinal laser photocoagulation stipulated that both medications are equivalent or equinumerous in controlling the pain produced by photocoagulation [**3**]. Keeping in mind the side effects of Ibuprofen, especially the gastrointestinal adverse reactions and the short period of drug administration, we could say that the first drug of choice should be Metamizole. It is now well established that inflammation plays a pathogenic role in age-related macular degeneration, diabetic retinopathy and diabetic macular edema, but clinical data demonstrating a therapeutic effect of NSAIDs for these diseases is limited and derived mostly from small, retrospective studies [**12**].

## Conclusions

- Metamizole induced a slight vasoconstriction (at the level of long posterior ciliary artery), probably COX1-mediated, for all doses used.

- Statistical significance of LPCA constriction was obtained only for the final moment (T4), after 5 minutes from the topical instillation and only for the Metamizole 5% and 6.66%.

- Metamizole administration did not increase the arterial diameter of LPCA in a dose dependent manner.

- The greatest concern related to the administration of metamizole is the risk of causing agranulocytosis [**1**], which is excluded in case of topical (ocular) administration.

- The high analgesic efficacy of metamizole, as well as its spasmolytic effect, makes it a very important pharmaceutical agent that could be used in the therapy of various eye disorders (dry eye syndrome, anterior pole inflammatory disorders, retinal vascular occlusions, or other retinal conditions) in humans and in animals.

**Conflict of interests **

The authors declare that they have no conflict of interests.

## References

[1] Jasiecka A, Maślanka T, Jaroszewski JJ (2014). Pharmacological Characteristics of Metamizole. Polish Journal of Veterinary Sciences.

[2] Barbosa de Araújo R, Zacharias LC, Marques de Azevedo B, Schmidt Giusti B, Pretti RC, Walter Y, Takahashi, Mário Luiz Ribeiro Monteiro (2015). Metamizole versus Placebo for Panretinal Photocoagulation Pain Control: A Prospective Double-Masked Randomized Controlled Study. International Journal of Retina and Vitreous.

[3] Macaferri Del Santo A, Maluf Auge R, Amaral Ferraz C (2016). Preemptive Analgesia of Metamizole versus Ibuprofen in Retinal Laser Photocoagulation. Revista Brasileira de Oftalmologia.

[4] Schug SA, Manopas A (2017). Update on the Role of Non-Opioids for Postoperative Pain Treatment. Best Practice & Research Clinical Anaesthesiology.

[5] Maślanka J, Rogosch JT (2012). Novel Bioactive Metabolites of Dipyrone (Metamizol). Bioorganic & Medicinal Chemistry.

[6] Vazquez E, Hernandez N, Escobar W, Vanegas H (2005). Antinociception Induced by Intravenous Dipyrone (Metamizol) upon Dorsal Horn Neurons: Involvement of Endogenous Opioids at the Periaqueductal Gray Matter, the Nucleus Raphe Magnus, and the Spinal Cord in Rats. Brain Research.

[7] Ahuja M, Dhake AS, Sharma SK, Majumdar DK (2008). Topical Ocular Delivery of NSAIDs. The AAPS Journal.

[8] Ricciotti E, FitzGerald GA (2011). Prostaglandins and Inflammation. Arteriosclerosis, Thrombosis, and Vascular Biology.

[9] Păunescu H, Coman OA, Coman L, Ghiţă I, Georgescu SR, Drăia F, Fulga I (2011). Cannabinoid System and Cyclooxygenases Inhibitors. Journal of Medicine and Life.

[10] Schalnus R (2003). Topical Nonsteroidal Anti-Inflammatory Therapy in Ophthalmology. Ophthalmologica. Journal International D’ophtalmologie. Topical Nonsteroidal Anti-Inflammatory Therapy in Ophthalmology. Ophthalmologica. Journal International D’ophtalmologie.

[11] De Paiva C, Coursey T (2004). Managing Sjögren’s Syndrome and non-Sjögren Syndrome dry eye with anti-inflammatory therapy. Clinical Ophthalmology.

[12] Schoenberger SD, Kim SJ (2013). Nonsteroidal Anti-Inflammatory Drugs for Retinal Disease. International Journal of Inflammation.

